# Towards the De Novo Design of HIV-1 Protease Inhibitors Based on Natural Products

**DOI:** 10.3390/biom11121805

**Published:** 2021-12-01

**Authors:** Ana L. Chávez-Hernández, K. Eurídice Juárez-Mercado, Fernanda I. Saldívar-González, José L. Medina-Franco

**Affiliations:** DIFACQUIM Research Group, Department of Pharmacy, School of Chemistry, Universidad Nacional Autónoma de México, Avenida Universidad 3000, Mexico City 04510, Mexico; anachavez3026@gmail.com (A.L.C.-H.); kaeuridice@gmail.com (K.E.J.-M.); fer.saldivarg@gmail.com (F.I.S.-G.)

**Keywords:** artificial intelligence, de novo design, fragment-based drug discovery, HIV-1 inhibitors, pseudo natural products

## Abstract

Acquired immunodeficiency syndrome (AIDS) caused by the human immunodeficiency virus (HIV) continues to be a public health problem. In 2020, 680,000 people died from HIV-related causes, and 1.5 million people were infected. Antiretrovirals are a way to control HIV infection but not to cure AIDS. As such, effective treatment must be developed to control AIDS. Developing a drug is not an easy task, and there is an enormous amount of work and economic resources invested. For this reason, it is highly convenient to employ computer-aided drug design methods, which can help generate and identify novel molecules. Using the de novo design, novel molecules can be developed using fragments as building blocks. In this work, we develop a virtual focused compound library of HIV-1 viral protease inhibitors from natural product fragments. Natural products are characterized by a large diversity of functional groups, many sp^3^ atoms, and chiral centers. Pseudo-natural products are a combination of natural products fragments that keep the desired structural characteristics from different natural products. An interactive version of chemical space visualization of virtual compounds focused on HIV-1 viral protease inhibitors from natural product fragments is freely available in the supplementary material.

## 1. Introduction

**Figure 1:**
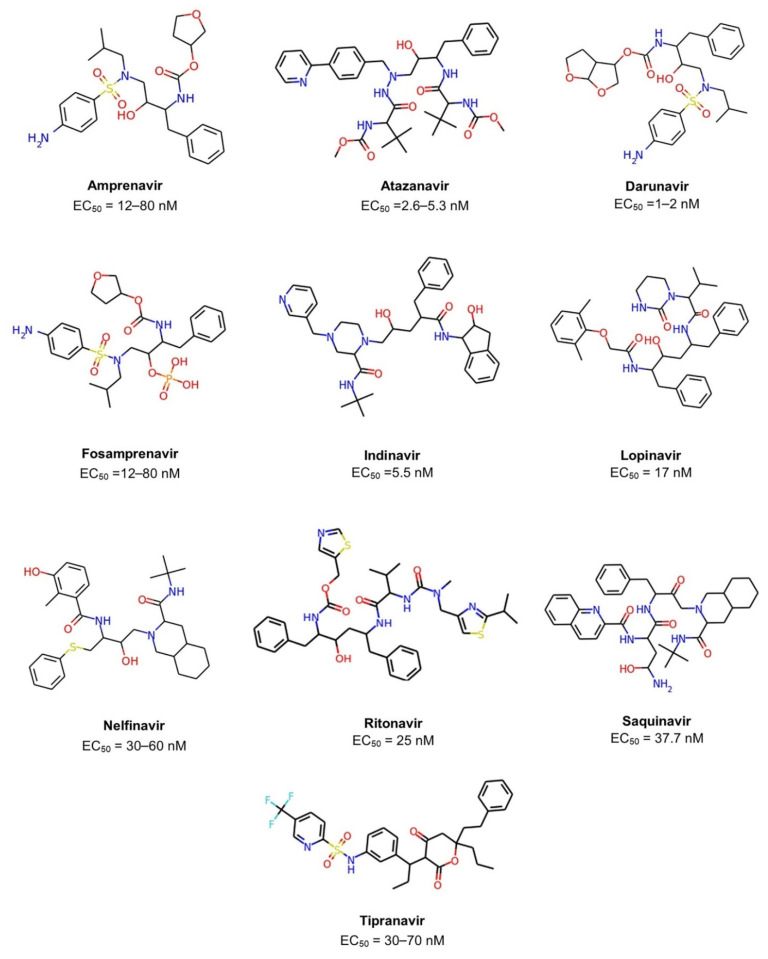
Chemical structures of ten FDA-approved HIV-1 protease inhibitors (Amprenavir, Atazanavir, Darunavir, Fosamprenavir, Indinavir, Lopinavir, Nelfinavir, Ritonavir, Saquinavir, Tipranavir). The EC50 is the concentration of drug required to produce 50% of the maximum possible effect.

The acquired immunodeficiency syndrome (AIDS) caused by the human immunodeficiency virus (HIV) is a major global public health concern. In 2020, the World Health Organization (WHO) reported that approximately 37.7 million people live with HIV out of 24.5 million from the African region. In 2020, 680,000 people died from HIV-related causes and 1.5 million people acquired it [1]. There is no definite treatment for AIDS. Therefore, it is necessary to collaborate to develop a treatment since the antiretroviral drugs currently approved by Food and Drug Administration (FDA) to clinical use only control AIDS and prevent HIV-1 transmission between individuals (Figure 1 and Table 1) [2,3,4].

Drug design and development demand many years of hard work and economic investment. Most drug candidates are prone to fail [5]. From 25,000 compounds that start in the laboratory, only 25 make it through preclinical testing to human testing, and just five of those reach the actual clinical use [6]. Computer-aided drug design (CADD) has contributed to yielding several drugs into the clinic, yet it has several challenges ahead [7]. Among the CADD methods, de novo design has gained relevance due to the diversity of structures generated by optimizing the algorithms used. From a methodological point of view, artificial intelligence as boosted the development and application of de novo design [5,8,9]. Notably, de novo design is a structure-based drug design method that benefits from the experimental information available of the binding sites of molecular targets.

The main goal of de novo design is to suggest novel molecular structures from scratch with desired activity on a pharmacological target and desired properties [10]. The new structures can be made using two general approaches: fragment-based and atom-based. The advantage of the fragment-based approach is that it narrows down the search in chemical space and maintains good chemical structure diversity [11,12,13]. Additionally, fragments form fewer interactions that should be able to bind to a greater number of sites on a greater number of proteins. Fragments are small (less than 20 heavy atoms) and typically soluble; they are likely to have better pharmaceutical properties as well as the new chemical compounds generated from them [14]. Over the last 20 years, four drugs from fragment-based drug discovery (FBDD) have been approved, and 40 compounds are currently in clinical trials [15].

Recently, de novo design and artificial intelligence have been combined to propose novel molecules for the treatment of SARS-CoV-2 based on HIV-1 protease and the approved drugs that inhibit this viral protease [8]. Another successful example of de novo design focusing on HIV research led to four molecules from a new compound library generated from the ZINC database [16]. Other approaches de novo design was based on enumerating libraries using chemical reactions [17,18] and are also promising to expand the epigenetic relevant chemical space [19].

The development of new chemical compounds using de novo design can begin from natural product-derived fragments. Natural products have been attractive chemical compounds because they are characterized by a larger number of sp^3^ carbon atoms, chiral centers (associated with structural complexity), the larger scaffold diversity, and functional groups, hence their relevance for use as building-blocks [20,21]. Indeed, larger structural complexity of small organic molecules has been associated with increased selectivity and drug-likeness. In previous studies, we showed that natural products cover regions of chemical space that have not yet been explored by synthetically accessible compounds and those with biological activity [22]. For this reason, natural products could be used as building-blocks to develop novel synthetic molecules or pseudo-natural products which combine the desired structural characteristics from different natural products [23].

The goal of this work was to develop a virtual focused compound library of HIV-1 protease inhibitors from natural products fragments through de novo design. The focused library was compared with two virtual libraries of HIV-1 protease inhibitors developed from commercially available fragment libraries that were used as reference. The commercial reference libraries were 4063 ChemDiv’s fragments (enriched with sp^3^ carbons) [24], and 4150 natural product fragments from Enamine [25]. The natural product fragments were built from the COlleCtion of Open NatUral producTs (COCONUT), the currently largest accessible database of natural products with more than 400,000 non-redundant compounds [26]. Of note, the novel chemoinformatics protocol presented herein is general and can be adapted to generate the compound libraries using de novo design, different molecular templates and molecular targets. Herein we focus on HIV-1 protease because of its current relevance in public health. Thus, we aim that the present work will contribute towards the research that leads to effective HIV treatments.

## 2. Materials and Methods

**Figure 2:**
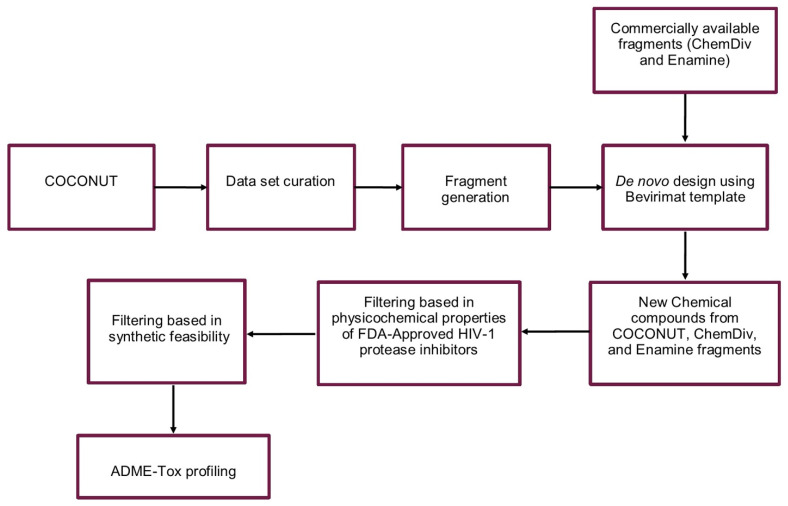
De novo design of the virtual focused compound libraries of HIV-1 viral protease inhibitors from natural product fragments (COCONUT) and commercially available fragments (ChemDiv and Enamine).

The virtual focused compound libraries of HIV-1 viral protease inhibitors from natural product fragments and two commercially available fragments libraries were developed using the protocol outlined in Figure 2.

### 2.1. Dataset Curation

The preparation of compounds, encoded in Simplified Molecular Input Line System (SMILES) [27], was performed using the open-source cheminformatics toolkit RDKit version 2021.03.3 [28], tool MolVS version 0.1.1 [29], and python programming language, version 3.7.10. Compounds with valence errors or any chemical element other than H, B, C, N, O, F, Si, P, S, Cl, Se, Br, and I were deleted. Stereochemistry information was removed because not all compounds in datasets have it defined. Compounds with multiple components were split, and the largest component was retained. The remaining compounds were neutralized and reionized to subsequently generate a canonical tautomer. Repeated compounds were deleted. To narrow down the search chemical space, physicochemical properties were computed: hydrogen bond donors (HBD), hydrogen bond acceptors (HBA), topological polar surface area (TPSA), number of rotatable bonds (RB), molecular weight (MW), and partition coefficient octanol/water (SlogP). Molecular compounds with the “rule of five” [30] and Veber [31] (MW ≤ 500, HBD ≤ 5, HBA ≤ 10, SlogP ≤ 5, TPSA ≤ 140, RB ≤ 10) were retained. Of note, despite the fact some of the fragments used in this work are generated from natural products (as illustrated in Figure 2), the type of molecules designed are small organic drug-like molecules.

### 2.2. Generation of Unique Fragments Using Retrosynthetic Rules

Fragment libraries were produced with the Retrosynthetic Combinatorial Analysis Procedure (RECAP) as implemented in RDKit. The RECAP algorithm [32] cleaves a molecule into fragments if this had any of the following bonds: amide, ester, amine, urea, ether, olefin, quaternary nitrogen, aromatic nitrogen–aliphatic carbon, lactam nitrogen–aliphatic carbon, aromatics carbon–aromatic carbon, and sulphonamide.

### 2.3. De Novo Design

**Figure 3:**
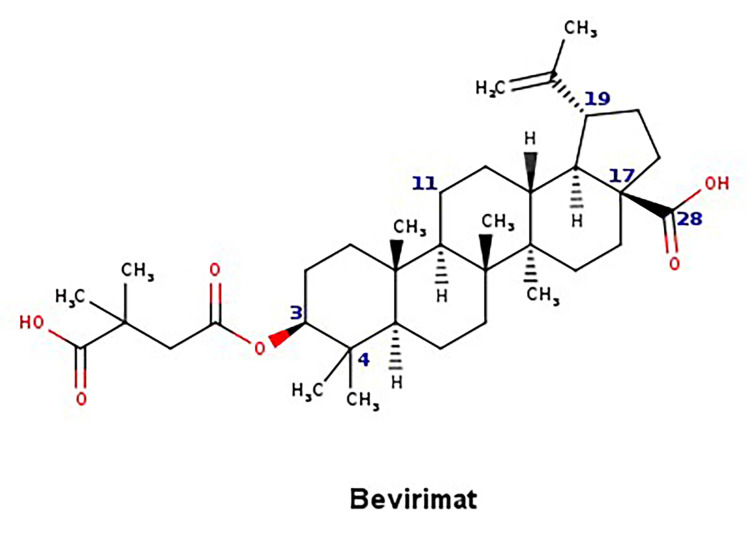
Chemical structure of bevirimat.

**Figure 4:**
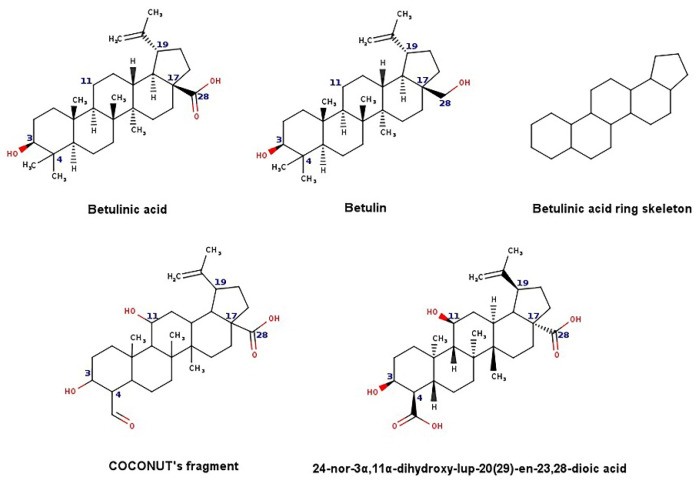
Chemical structures of betulinic acid, betulin, cyclic system skeleton derived from betulinic acid, COCONUT’s fragment with betulinic acid ring skeleton derived from the 24-nor-3α,11α-dihydroxy-lup-20(29)-en-23,28-dioic acid.

**Figure 5:**
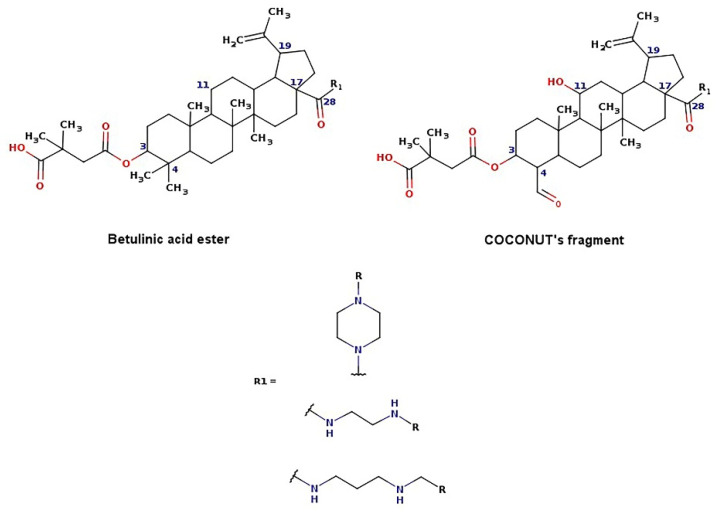
Template for building new chemical compounds similar to bevirimat using the ester of betulinic acid to ChemDiv fragments and Enamine fragments, and the ester of COCONUT’s fragment derived from 24-nor-3α,11α-dihydroxy-lup-20(29)-en-23,28-dioic acid.

The new chemical structures were built based on the template previously proposed by Zhao et al. developed from the structure-activity relationship (SAR) analysis for the optimization of bevirimat (Figure 3), a compound derived from betulinic acid (Figure 4) [33]. Bevirimat [34,35] is a compound in clinical trials that targets the Gag polyprotein inhibiting the action of HIV protease at its the last cleavage event of the capsid protein and spacer peptide 1 (CA-SP1) [36,37]. The template proposed for building new chemical compounds related to bevirimat is shown in Figure 5.

New molecules were generated using the Python programming language and the toolkit RDKit [28], following the protocol described for Saldívar-González et al. to enumerate chemical libraries [18]. We used COCONUT fragments with a cyclic system skeleton similar to betulinic acid, a hydroxyl group attached to carbon 3, and a carboxylic acid group attached to carbon 17, as shown in Figure 4. The COCONUT’s fragment selected was derived from 24-nor-3α,11α-dihydroxy-lup-20(29)-en-23,28-dioic acid (COCONUT ID: CNP0243494 or Reaxys ID: 6547020). Betulinic acid was used to build new chemical compounds from ChemDiv fragments and Enamine fragments because there were no fragments of cyclic system skeleton derived from betulinic acid or analogous triterpenes.

Chemical reactions were represented in SMIRKS, a hybrid notation of SMILES and SMARTS (SMILES Arbitrary Target Specification). Reaction 1, esterification, was made between triterpene alcohol and 2,2-dimethyl succinic acid using SMIRKS 1, as shown in Table 2. Reaction 2, amidation, was built from the carboxyl group attached to carbon 17 as shown in Figure 4 using fragments attached to piperazine, 1,3-diaminoethane, and 1,3-diaminopropane find in COCONUT fragments, ChemDiv fragments, and Enamine fragments. The SMIRKS 2.1–2.3 were used in reaction 2 and shown in Table 2. The compounds and fragments were selected using the functional groups in SMARTS notation described in Table 3. Newly generated chemical structures with valence errors were removed. Canonical SMILES were generated, and duplicate molecules were deleted. 

### 2.4. Structural Diversity and Complexity

The structural diversity of the new chemical compounds generated was evaluated to compute the median value of the distribution of the pairwise similarity values generated with the Tanimoto coefficient for Morgan fingerprint with radius 2 (Morgan2, 1024-bits) [38] and Molecular ACCes System (MACCS) Keys (166-bits) [39].

### 2.5. Chemical Space Visualization

The chemical space visualization was done using two methods, principal component analysis (PCA) based on physicochemical properties and the Tree MAP (TMAP) algorithm based on molecular fingerprints [40,41].

PCA is a linear dimensionality reduction technique to transform data with many dimensions into a lower dimensional space and preserve the different relationships between the data points as much as possible [42]. PCA was generated from six physicochemical properties (MW, HB, HBA, SlogP, TPSA, and RB).

TMAP allows the visual representation of many chemical compounds through the distance between the clusters and the cluster’s detailed structure through Local Sensitive Hashing (LSH) forest data structure, enabling c-approximate k-nearest neighbors (k-NN). Morgan fingerprints for chemical compounds were encoded using the MinHash algorithm. The number of nearest-neighbors, k = 50, and the factor used by the augmented query algorithm, kc = 10, were used to develop the TMAP graphs. Morgan fingerprints with radius 2 (Morgan2, 1024-bits) were generated to generate TMAP graphs [38]. Applications of TMAP for chemical space visualization of other compound datasets have been reported [43,44].

### 2.6. Filtering of the New Chemical Compounds Generated

To narrow down the search in chemical space and set the conditions for the newly generated compounds, physicochemical properties were computed for libraries generated and FDA-approved HIV-1 protease inhibitors (Table 1 and Figure 1). The maximum values of the physicochemical properties obtained from the HIV-1 protease inhibitors was HBD ≤ 6, HBA ≤ 13, SlogP ≤ 6.7, MW ≤ 720.30, TPSA ≤ 174.60, and RB ≤ 17 (Table 4). Molecules with at least four rules were retained. SlogP strictly must be complied. These sets of properties and values were used as a heuristic rule that is slightly less stringent than the Lipinski and Veber rules [30,31].

### 2.7. Synthetic Feasibility

The complexity of the compounds generated was estimated using the synthetic accessibility score (SAscore) previously reported [45]. The SAscore implemented in this work is the difference between fragment score and complexity penalty. The fragment score captures common structural features in a large number of already synthesized molecules (934,046 representative molecules from the PubChem). Molecules are fragmented using extended connectivity fragments (ECFP_4# fragments), and the fragment score is calculated as a sum of contributions of all fragments in the molecule divided by the number of fragments in the molecule. The fragment frequency is related to their synthetic accessibility, and hence easy-to-prepare substructures are present in molecules quite often. The complexity score is calculated as the sum of ring complexity (ring bridge atoms and spiro atoms), the number of stereocenters, large rings (ring size greater than eight, molecular complexity increases), and molecule size. The SAscore was calculated for the virtual focused libraries of HIV-1 viral protease inhibitors generated, and two reference datasets of FDA-approved drugs, and FDA-approved HIV-1 protease inhibitors [46]. The SAscore was calculated using the Python script published by Ertl and Schuffenhauer [45].

### 2.8. ADME-Tox Profiling

Absorption, distribution, metabolism, excretion, and toxicity (ADME-Tox) properties of virtual focused libraries of HIV-1 viral protease inhibitors generated were calculated using the SwissADME server [47] and the pkCSM-pharmacokinetics server [48]. The ADME-Tox properties of FDA-approved drugs were also computed as reference. The SwissADME server was used to compute descriptors associated with absorption and metabolism. The pkCSM-pharmacokinetics server was used to compute descriptors associated with absorption, distribution, excretion, and toxicity. The evaluation of descriptors related to ADME-Tox properties was computed as previously described [49]. The descriptors calculated were absorption broken down into solubility, Silico-IT LogSw; lipophilicity, consensus LogPo/w, and human intestinal absorption (HIA). The blood-brain barrier (BBB) permeability, P-glycoprotein substrate, P-glycoprotein I inhibitor, and P-glycoprotein II (take binary values: yes/no) for distribution. Inhibition of five main cytochrome enzymes (CYP-1A2, CYP-2C19, CYP-2C9, CYP-2D6, CYP-3A4) for metabolism (take binary values: yes/no). Total clearance log (mL/min/kg) to excretion. The hERG I/II inhibition, AMES toxicity, and hepatotoxicity to toxicity (take binary values: yes/no).

## 3. Results and Discussion

As mentioned in the Introduction and Methods sections, new chemical compounds were built from two commercially available libraries: 4063 ChemDiv fragments enriched with sp^3^ carbons, 4160 Enamine natural products fragments, and 184,769 COCONUT fragments computationally generated in house. The total number of molecules generated were: 1534 from COCONUT’s fragments, 62 molecules from ChemDiv fragments, and 11 molecules from Enamine fragments. Fragments attached to 1,3-diaminopropane were not found in ChemDiv and Enamine’s fragment collections. Similarly, fragments attached to 1,2-diaminoethane were not found in Enamine fragments. 

### 3.1. Structural Diversity

The median of similarity generated using Morgan2 and MACCS keys fingerprints are shown in brackets, respectively, and described in Table S1 in the supplementary material. FDA-approved drugs (0.096, 0293) and FDA-approved HIV-1 protease inhibitors (0.253, 0.558) were the most diverse datasets, following by compounds derived from COCONUT fragments (0.605, 0.817), ChemDiv fragments (0.676, 0.821), and Enamine fragments (0.682, 0.823). Compounds computationally generated from fragment datasets were less diverse because these datasets are focused on bevirimat-like compounds. 

### 3.2. Chemical Space Visualization

**Figure 6:**
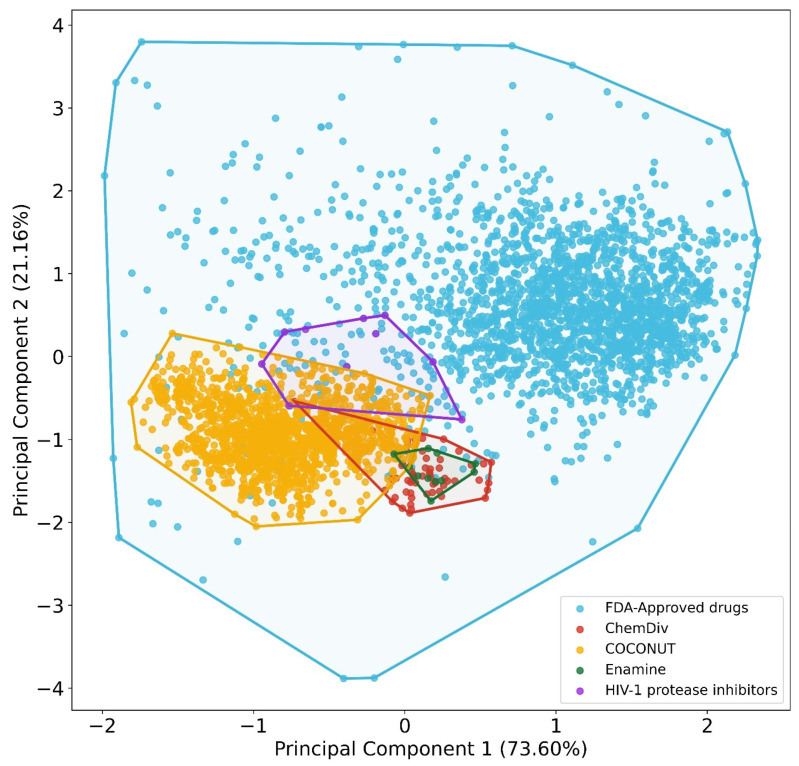
Chemical space visualization of the virtual focused compound library of HIV-1 viral protease inhibitors from natural product fragments and two compound reference libraries using PCA based on physicochemical properties. Compound reference libraries represented in colors: FDA-approved drugs (blue) and FDA-approved HIV-1 protease inhibitors (purple). Likewise. for new chemical compounds generated from COCONUT (orange), ChemDiv (red), and Enamine (green) fragment libraries.

A visual representation of the chemical space based on physicochemical properties (MW, HB, HBA, SlogP, TPSA, and RB, as stated in the Methods Section 2.5) using PCA is shown in Figure 6. Principal component 1 recovered 73.6% of the variance, and principal component 2 recovered 21.2% of the variance. The accumulated variance recovered by the first two principal components represented in Figure 6 was 94.8%. In this chemical space visualization, the compounds generated from the three fragment libraries are within the space of physicochemical properties of FDA-approved drugs. Likewise, some compounds generated from COCONUT fragments had physicochemical properties similar to FDA-approved HIV-1 protease.

To quantitatively define which dataset is the most diverse, coverage space obtained by convex hull analysis derived from PCA was computed for each dataset (Figure S1). The convex hull is defined as the minimum convex polygon so that the point set is either inside this polygon or at its border [50,51]. The convex hull area computed were for FDA-approved drugs (737.59), HIV-1 protease inhibitors (1.11), compounds from COCONUT’s fragments (3.18), compounds from ChemDiv’s fragments (0.79), and compounds from Enamine fragments (0.18). The outcome of this analysis was similar to the results of the structural diversity analysis based on fingerprints (Section 3.1): reference datasets were more diverse than the new chemical compounds generated from fragments datasets. The new chemical compounds derived from COCONUT fragments were the most diverse, followed by new chemical compounds derived from ChemDiv and Enamine fragments.

**Figure 7:**
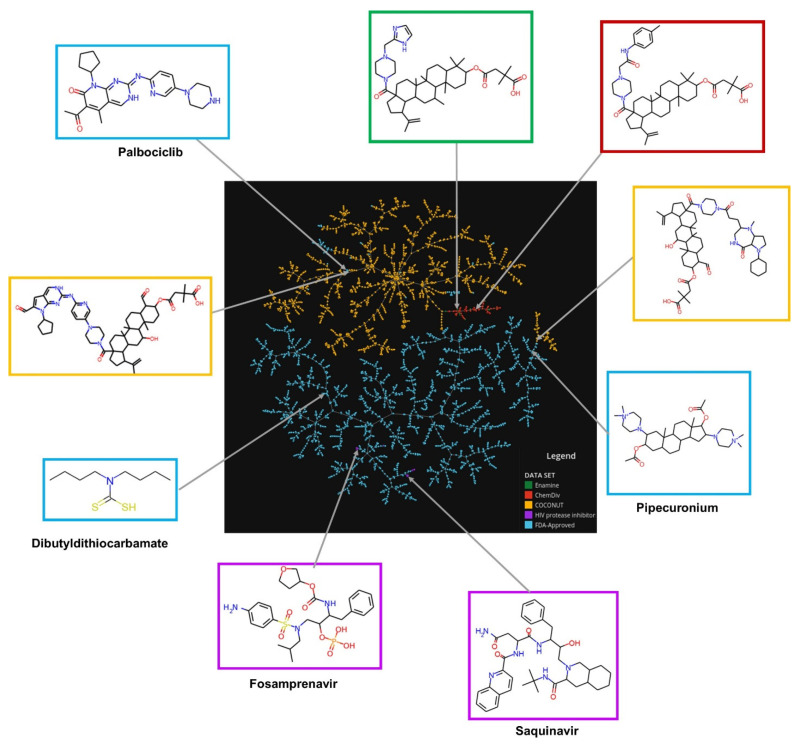
Chemical space visualization of the virtual focused compound library of HIV-1 viral protease inhibitors from natural product fragments and two compound reference libraries using TMAP based on molecular fingerprints. Compounds reference libraries represented in colors: FDA-approved drugs (blue), and FDA-approved HIV-1 protease inhibitors (purple). Likewise, for new chemical compounds generated from COCONUT (orange), ChemDiv (red), and Enamine (green) fragment libraries. The interactive version is available at https://figshare.com/s/ceb58d58e8f5585ce67e (accessed on 5 November 2021).

The visual representation of the chemical space based on molecular fingerprint using the TMAP algorithm is shown in Figure 7. An interactive version of the TMAP is available at https://figshare.com/s/ceb58d58e8f5585ce67e (accessed on 5 November 2021). The chemical structures of new chemical compounds generated were very different in comparison with FDA-approved drugs and FDA-HIV-1 protease inhibitors. The chemical structures of the new compounds generated from ChemDiv and Enamine fragments were very similar compared to compounds derived from COCONUT fragments. In some cases, the chemical structures of compounds generated from COCONUT’s fragments were very similar to some FDA-approved drugs, for instance, palbociclib and pipecuronium. In these cases where there are not commercially available fragments like COCONUT’s fragments could be used palbociclib and pipecuronium.

### 3.3. Compound Filtering Based on Physicochemical Properties

**Figure 8:**
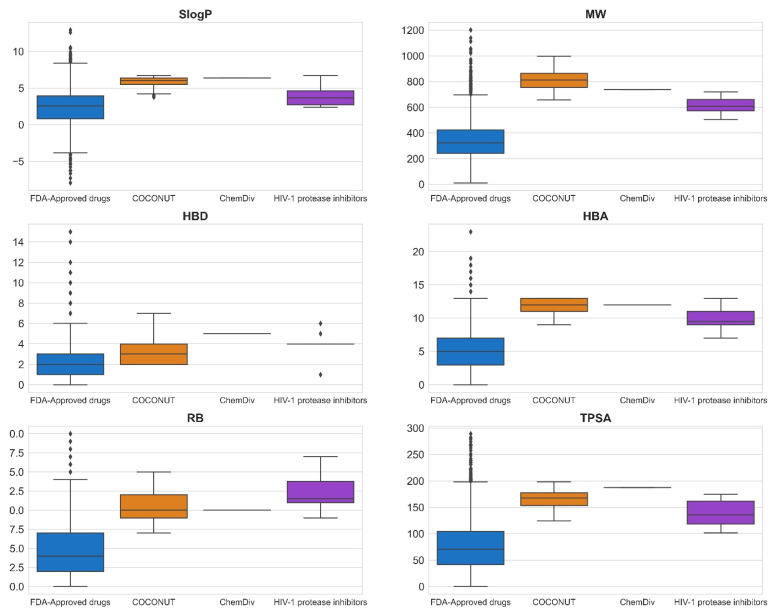
Box-whisker plots of physicochemical properties of FDA-approved drugs (blue), FDA-approved HIV-1 protease inhibitors (purple), and new chemical compounds generated from COCONUT (orange) and ChemDiv (red) fragment libraries after applying physicochemical properties filtering. Black diamonds represent outliers.

Figure 8 shows box-whisker plots of physicochemical properties after applying the empirical rules proposed (Section 2.6). The summary of descriptive statistics is shown in Tables S2–S7 in the supplementary material. 352 compounds generated from COCONUT fragments (20%) and 1 compound generated from ChemDiv fragments were retained (2%), and compounds generated from Enamine fragments were not retained (0%). Based on the properties’ distribution shown in the box-whisker plots, the physicochemical properties of compounds generated from COCONUT fragments, ChemDiv fragments, and Enamine fragments were different regarding FDA-approved HIV-1 protease inhibitors and FDA-approved drugs. 

The physicochemical properties calculated for datasets were: SlogP ≤ 12.94, MW ≤ 1201.84, RB ≤ 20, TPSA ≤ 286.50, HBA ≤ 23, HBD ≤ 15 for FDA-approved drugs; SlogP ≤ 6.70, MW ≤ 720.31, RB ≤ 17, TPSA ≤ 174.56, HBA ≤ 13, HBD ≤ 6 for FDA-approved HIV-1 protease inhibitors; SlogP ≤ 6.69, MW ≤ 998.63, RB ≤ 15, TPSA ≤ 198.54, HBA ≤ 13, HBD ≤ 7 for compounds generated from COCONUT fragments, and SlogP = 6.4, MW = 737.47, RB = 10, TPSA = 187.47, HBA = 12, HBD = 5 for the compound generated from ChemDiv’s fragments. The SlogP, RB, and HBA values of compounds generated from COCONUT fragments and ChemDiv fragments were less than FDA-approved HIV-1 protease inhibitors. HBA values were equal or less than FDA-approved HIV-1 protease inhibitors. The SlogP values of compounds derived from Enamine fragments were larger than FDA-approved HIV-1 protease inhibitors as shown in Figure S2; accordingly, no compound was retained. The MW, TPSA, and HBD values of compounds generated from COCONUT fragments were larger than for FDA-approved HIV-1 protease inhibitors and less than for FDA-approved drugs. As mentioned above Ganesan [52], natural products that violate the Lipinsky rules remain largely compliant in terms of log P and HBD. He considers that “nature has learned to maintain low hydrophobicity and intermolecular H-bond donating potential when it needs to make biologically active compounds with high molecular weight and a large number of rotatable bonds”. In drugs, the molecules that exceed HBD 5 or HBA 10 the majority are natural product-related [53].

### 3.4. Filtering Based on Synthetic Feasibility

**Figure 9:**
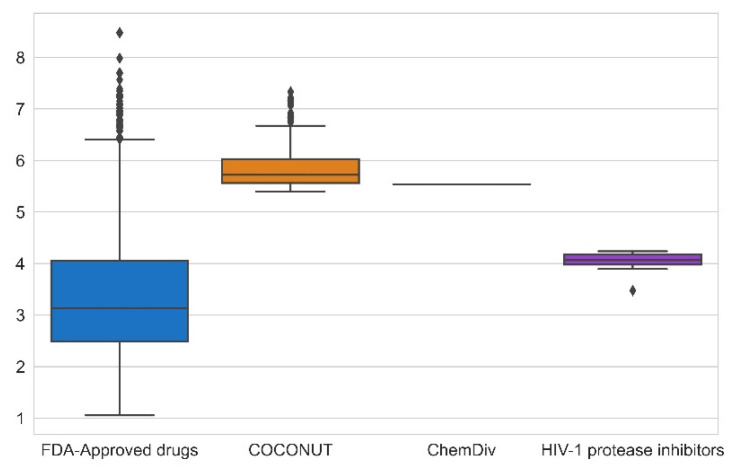
Box-whisker plot of synthetic feasibility calculated for FDA-approved drugs (blue), FDA-approved HIV-1 protease inhibitors (purple), and new chemical compounds generated from COCONUT fragments (orange) and ChemDiv (red) fragments with physicochemical properties like FDA-approved HIV-1 protease inhibitors. Black diamonds represent outliers.

The synthetic feasibility was computed for FDA-approved drugs, FDA-approved HIV-1 protease inhibitors, and compounds generated from COCONUT and ChemDiv fragments with physicochemical properties like FDA-approved HIV-1 protease inhibitors. Figure 9 summarizes the results of synthetic feasibility. Molecules with a low SAscore value < 6 are easily synthetically accessible [45]. A total of 97% FDA-approved drugs had SAscore < 6, and FDA-approved HIV-1 protease inhibitors had SAscore ≤ 4.24. Similarly, 75% of compounds generated from COCONUT fragments had SAscore ≤ 6.03 and the compound generated from ChemDiv had SAscore = 5.54. Although, compounds generated from COCONUT fragments had 5.50 ≤ SAscore ≤ 6.03, still in recommended range so that can be synthetically accessible; moreover, the high SAscore, in compounds generated regarding FDA-approved HIV-1 protease inhibitors, was influenced by the ten stereocenters of betulinic acid and 24-nor-3α,11α-dihydroxy-lup-20(29)-en-23,28-dioic acid. Considering that these stereocenters do not have to be generated within the organic synthesis, the SAscore value would be lower.

### 3.5. ADME-Tox Profiling

The ADME-Tox profiling was computed for 251 compounds generated from COCONUT fragments and 1 compound generated from ChemDiv fragments with physicochemical properties like FDA-approved HIV-1 protease inhibitors and estimated as easy synthesizable (i.e., SAscore ≤ 6). Similarly, ADME-Tox profiling was computed for FDA-approved drugs and FDA-approved HIV-1 protease inhibitors.

#### 3.5.1. Absorption

**Figure 10:**
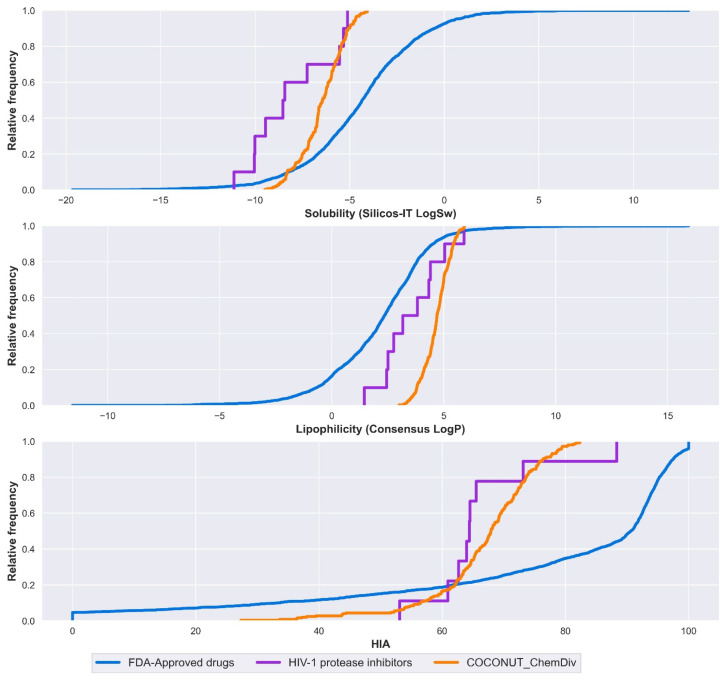
Distribution curve of solubility, lipophilicity, and HIA. Colors represent compounds: new chemical compounds generated from COCONUT fragments and ChemDiv fragments with physicochemical properties like FDA-approved HIV-1 protease inhibitors and easily synthetically accessible (orange), FDA-approved drugs (blue), FDA-approved HIV-1 protease inhibitors (purple). Solubility is expressed in the percentage of Silicos-IT LogSw, and lipophilicity is expressed in the percentage of consensus LogP.

Solubility, lipophilicity, and HIA are summarized in Figure 10 and Tables S9–S11 in the supplementary material. Solubility was expressed by Silicos-IT LogSw and lipophilicity was expressed by consensus LogP. Silicos-IT LogSw and consensus LogP were computed with the SwissADME server. Percentage of HIA was computed with the pkCSM-pharmacokinetics server. 

Median values for solubility, lipophilicity, and HIA are described below. FDA-approved drugs had consensus LogP = 2.36, Silicos-IT LogSw = −4.34, HIA = 90.6%. FDA-approved HIV-1 protease inhibitors had consensus LogP = 3.50, Silicos-IT LogSw = −8.49, HIA = 64.4%. Compounds derived from COCONUT and ChemDiv had consensus LogP = 4.70 and Silicos-IT LogSw = −6.45, HIA = 67.9%. 

New drug candidates have poor water solubility, and it is often the result of highly lipophilic compounds. Log P < 2, the crystal lattice becomes the main determining factor for solubility. LogP values above 2, the lipophilicity is the main factor [54]. FDA-approved HIV-1 protease inhibitors were highly soluble, followed by compounds derived from COCONUT and ChemDiv fragments, both had Log P > 2; in this case, solubility is strongly influenced by lipophilicity. Contrary to FDA-approved drugs that had Log P close to 2 and were less soluble, solubility mainly depends on the crystal lattice. Compounds derived from COCONUT and ChemDiv fragments had higher HIA in comparison to FDA-approved HIV-1 protease inhibitors.

#### 3.5.2. Distribution

**Figure 11:**
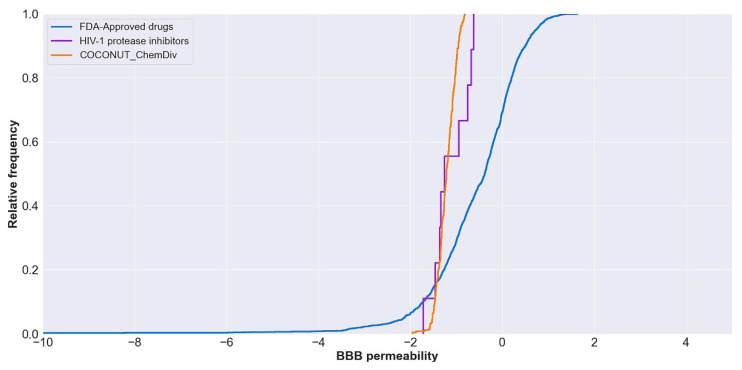
Distribution curve of BBB permeability. Colors represent compounds: new chemical compounds generated from COCONUT fragments and ChemDiv fragments with physicochemical properties like FDA-approved HIV-1 protease inhibitors and easily synthetically accessible (orange), FDA-approved drugs (blue), FDA-approved HIV-1 protease inhibitors (purple). The BBB permeability of FDA-approved drugs was between −34 and 2.

The relative frequency of BBB permeability is described in Figure 11. The median value of BBB permeability was −0.38 for FDA-approved drugs; −1.21 for compounds generated from COCONUT and ChemDiv fragments, and −1.25 for FDA-approved HIV-1 protease inhibitors. Compounds generated from COCONUT and ChemDiv fragments had similar BBB permeability.

**Figure 12:**
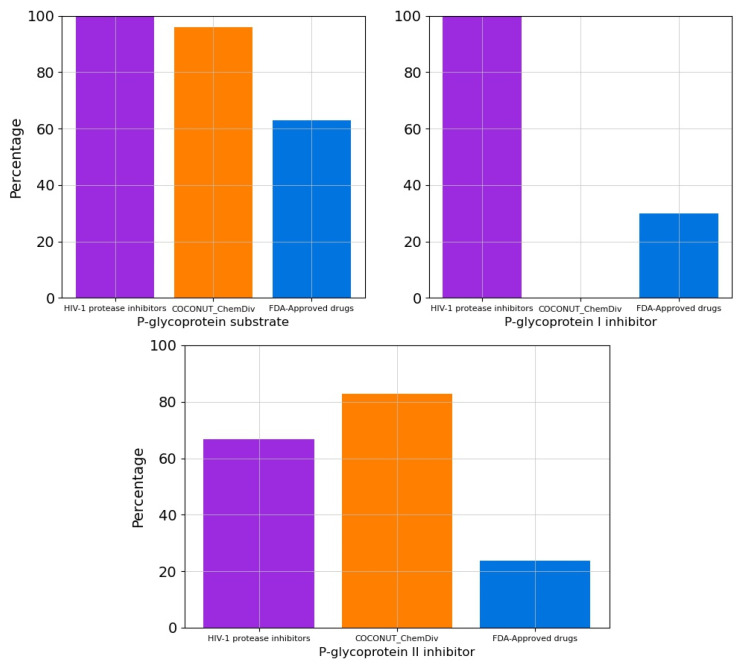
Percentage of compounds that are P-glycoprotein substrate, P-glycoprotein I inhibitor, and P-glycoprotein II inhibitor. Colors represent compounds: new chemical compounds generated from COCONUT fragments and ChemDiv fragments with physicochemical properties like FDA-approved HIV-1 protease inhibitors and easily synthetically accessible (orange), FDA-approved drugs (blue), FDA-approved HIV-1 protease inhibitors (purple).

The percentage of compounds that are P-glycoprotein substrate, P-glycoprotein I inhibitor, and P-glycoprotein II inhibitor were summarized in Figure 12 and Table S13 in the supplementary material. All FDA-approved HIV-1 protease inhibitors and 96% of compounds generated from COCONUT and ChemDiv fragments were P-glycoprotein substrates. Similarly, 66.67% of HIV-1 Approved protease inhibitors and 82.9% of compounds generated from COCONUT and ChemDiv fragments were P-glycoprotein II inhibitors. Whereas no compounds generated from COCONUT and ChemDiv fragments were P-glycoprotein I inhibitors, against 100% FDA-approved HIV-1 proteases inhibitors were P-glycoprotein I inhibitors.

#### 3.5.3. Metabolism

**Figure 13:**
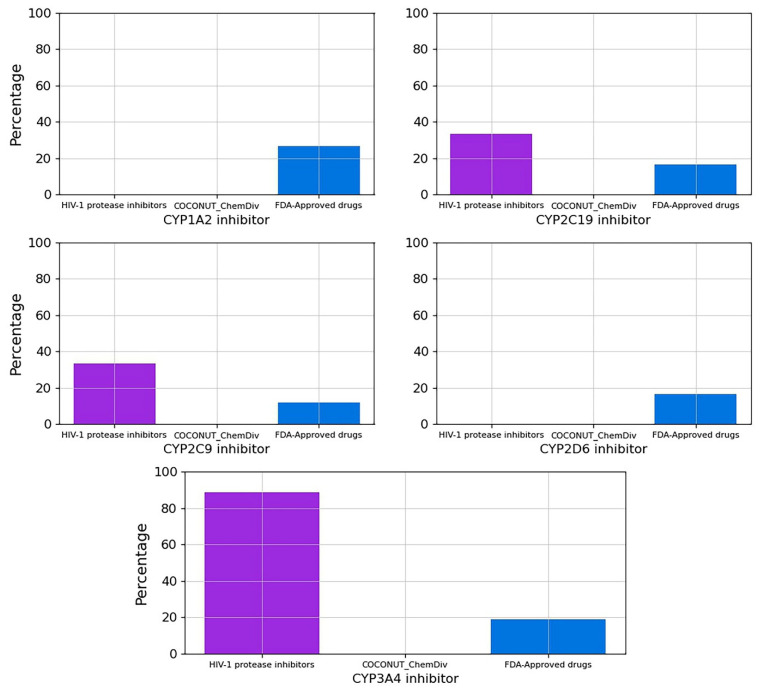
Percentage of compounds that inhibit the main cytochromes, CYP1A2, CYP2C19, CYP2C9, CYP2D6, CYP3A4. Colors represent compounds: new chemical compounds generated from COCONUT fragments and ChemDiv fragments with physicochemical properties like FDA-approved HIV-1 protease inhibitors and easily synthetically accessible, FDA-approved drugs (blue), FDA-approved HIV-1 protease inhibitors (purple).

The percentage of compounds CYP1A2, CYP2C19, CYP2C9, CYP2D6 and CYP3A4 inhibitors is described in Figure 13 and Table S14 in the supplementary material. No compounds generated from COCONUT and ChemDiv fragments were CYP1A2, CYP2C19, CYP2C9, CYP2D6 and CYP3A4 inhibitors. FDA-approved HIV-1 inhibitors were not CYP1A2 and CYP2D6 inhibitors similar to compounds generated from COCONUT and ChemDiv fragments. Whereas for FDA-approved HIV-1protease inhibitors, 89% were CYP3A4 inhibitors, and 33% were CYP2C19 and CYP2C9 inhibitors.

#### 3.5.4. Excretion

**Figure 14:**
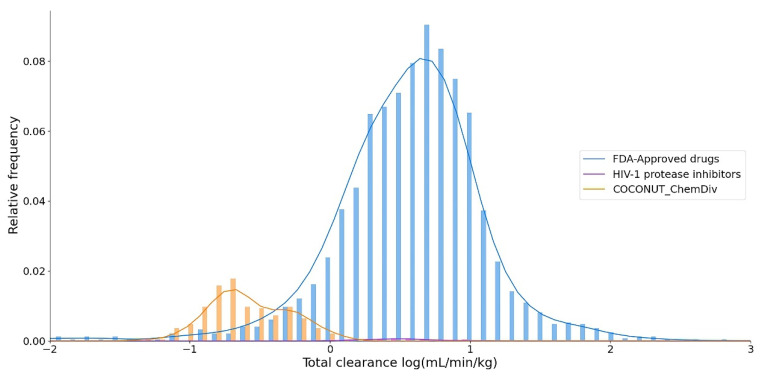
Distribution curve of the total clearance. Colors represent compounds: new chemical compounds generated from COCONUT fragments and ChemDiv fragments with physicochemical properties like FDA-approved HIV-1 protease inhibitors and easily synthetically accessible (orange), FDA-approved drugs (blue), FDA-approved HIV-1 protease inhibitors (purple).

Clearance quantitates the irreversible removal of a drug from the measured matrix, generally, blood or plasma [55]. The total clearance logarithm expressed in units of (mL/min/Kg) is shown in Figure 14. The summary of descriptive statistics is shown in Table S15 in the Supplementary Materials. The median values of the total clearance logarithm were 0.591 for FDA-approved drugs; 0.494 for FDA-approved HIV-1 protease inhibitors, and −0.618 for compounds derived from COCONUT and ChemDiv fragments. The total clearance of FDA-approved HIV-1 protease inhibitors (0.20 ≤ total clearance ≤ 0.94) was similar to 75% FDA-approved drugs (0.27 ≤ total clearance ≤ 0.85). Whereas the total clearance of compounds generated from COCONUT and ChemDiv fragments (−1.34 ≤ total clearance ≤ 0.13) was similar to 25% FDA-approved drugs (-13.94 ≤ total clearance ≤ 0.27). The total clearance of compounds derived from COCONUT and ChemDiv fragments and FDA-approved HIV-1 inhibitors were different.

#### 3.5.5. Toxicity

**Figure 15:**
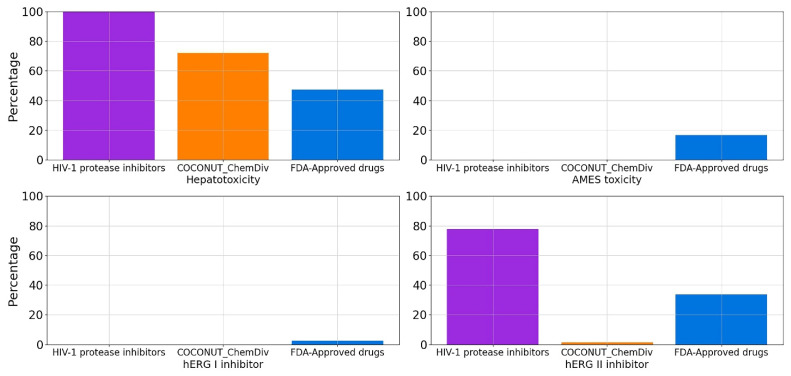
Percentage of compounds that are hERG I inhibitor, hERG II inhibitor, hepatotoxicity, and toxicity in AMES test in silico. Colors represent compounds: new chemical compounds generated from COCONUT fragments and ChemDiv fragments with physicochemical properties like FDA-approved HIV-1 protease inhibitors and easily synthetically accessible (orange), FDA-approved drugs (blue), FDA-approved HIV-1 protease inhibitors (purple).

Percentage of compounds from datasets that are hERG I inhibitor, hERG II inhibitor, hepatotoxicants (hepatotoxicity), and carcinogens (positive in AMES test) were described in Figure 15 and Table S16 in the supplementary material. FDA-approved HIV-1 protease inhibitors and compounds generated from COCONUT and ChemDiv fragments were not carcinogens. However, 77.22% of compounds derived from COCONUT and ChemDiv fragments were hepatotoxicants, lower than FDA-approved HIV-1 protease inhibitors (100%), and higher than FDA-approved drugs (47.42%). A total of 100% and 98.81% of compounds generated from COCONUT and ChemDiv fragments were not hERG I/II inhibitors, respectively.

## 4. Conclusions

We developed an HIV-1 virtual focused library using de novo design based on enumerated libraries of compounds from fragment libraries. The fragments library in-house was built from the COCONUT database, the currently largest accessible database of natural products. Using bevirimat as template, 251 out of 1534 compounds generated from COCONUT fragments, had physicochemical properties like FDA-approved HIV-1 protease inhibitors and were estimated as easy synthesizable.

Compounds generated from COCONUT fragments were more diverse than compounds generated from ChemDiv and Enamine fragments, based on chemical structure and physicochemical properties. Visual representation of the chemical space based on TMAP showed that some compounds generated from COCONUT fragments had chemical structures similar to FDA-approved drugs, such as palbociclib and pipecuronium.

ADME/Tox profiling showed that compounds generated from COCONUT fragments had adsorption (solubility and lipophilicity) and distribution (BBB permeability, P-glycoprotein substrate, and P-glycoprotein II inhibitor) similar to FDA-approved HIV-1 protease inhibitors. Concerning estimations of metabolism, no compounds generated from COCONUT fragments were CYP1A2, CYP2C19, CYP2C9, CYP2D6, and CYP3A4 inhibitors. As per excretion, the total clearance of compounds derived from COCONUT fragments and FDA-approved HIV-1 inhibitors were different, but similar to FDA-approved drugs. Compounds derived from COCONUT fragments were predicted to be no inhibitors of hERG I/II, like 97.7% and 66.4% of FDA-approved drugs, respectively. Compounds derived from COCONUT fragments were predicted to be no carcinogens. 

The 251 compounds derived from COCONUT fragments with physicochemical properties like FDA-approved HIV-1 protease inhibitors, estimated as easy synthesizable, and good ADME/Tox profiling can be used in future analysis such as virtual screening to select candidates to test in biological assays. The next logical perspective of this project that this is beyond the scope of this manuscript is to conduct the chemical synthesis and experimental screening of selected compounds.

The protocol presented in this work is general and can be used to build other chemical compounds like bevirimat or other maturation inhibitors of HIV-protease. Notably, the code used for generated new chemical compounds from chemical fragments is freely available (see Data Availability statement). This can be achieved from the SMARTS and SMIRKS proposed to filter functional groups and build new chemical compounds.

## Figures and Tables

**Table 1:** FDA-approved HIV-1 protease inhibitors which will be used as a reference for the de novo design of the new chemical compounds. ^a^ Fosamprenavir is the phosphate ester prodrug of amprenavir.

Generic Name	Brand Name	EC_50_ [3]	FDA Approval
Amprenavir	Agenerase	12–80 nM	1999
Atazanavir	Reyataz	2.6–5.3 nM	2003
Darunavir	Prezista	1–2 nM	2006
Fosamprenavir ^a^	Lexiva	12–80 nM	2003
Indinavir	Crixivan	5.5 nM	1996
Lopinavir	Kaletra	17 nM	2000
Nelfinavir	Viracept	30–60 nM	1997
Ritonavir	Norvir	25 nM	1996
Saquinavir	Invirase	37.7 nM	1995
Tipranavir	Aptivus	30–70 nM	2005

**Table 2:** SMIRKS used for building the new chemical compounds from natural products fragments.

Description	Scheme
Reaction 1	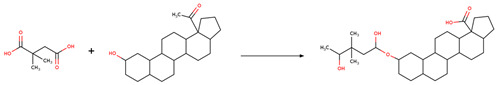
SMIRKS 1	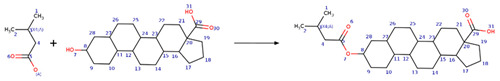 [#6:1][#6;A;X4:3]([#6:2])[#6:4]-[#6:5]([#8;A])=[O:6].[#8:7]-[#6:8]-1-[#6:9]-[#6:10]-[#6:11]-2-[#6:27](-[#6:26]-[#6:25]-[#6:24]-3-[#6:23]-4-[#6:22]-[#6:21][C:20]5([#6:19]-[#6:18]-[#6:17]-[#6:16]5-[#6:15]-4-[#6:14]-[#6:13]-[#6:12]-2-3)[#6:29](-[#8:31])=[O:30])-[#6:28]-1>>[#6:2][#6;A;X4:3]([#6:1])[#6:4]-[#6:5](=[O:6])-[#8:7]-[#6:8]-1-[#6:9]-[#6:10]-[#6:11]-2-[#6:27](-[#6:26]-[#6:25]-[#6:24]-3-[#6:23]-4-[#6:22]-[#6:21][C:20]5([#6:19]-[#6:18]-[#6:17]-[#6:16]5-[#6:15]-4-[#6:14]-[#6:13]-[#6:12]-2-3)[#6:29](-[#8:31])=[O:30])-[#6:28]-1
Reaction 2.1	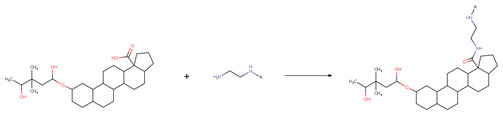
SMIRKS 2.1	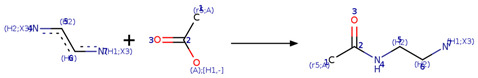 [#7;H1;X3:7][#6H2:6][#6;H2:5][#7;H2;X3:4].[#6;A;r5:1][#6:2]([#8;A;H1,-])=[O:3]>>[#6;A;r5:1][#6:2](=[O:3])-[#7:4]-[#6;H2:5]-[#6;H2:6]-[#7;H1;X3:7]
Reaction 2.2	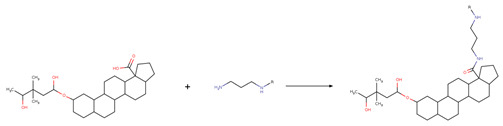
SMIRKS 2.2	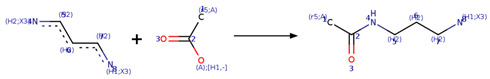 [#7;H1X3:8][#6H2:7][#6H2:6][#6H2:5][#7;H2X3:4].[#6;A;r5:1][#6:2]([#8;A;H1,-])=[O:3]>>[#6;A;r5:1][#6:2](=[O:3])-[#7:4]-[#6H2:5]-[#6H2:6]-[#6H2:7]-[#7;H1X3:8]
Reaction 2.3	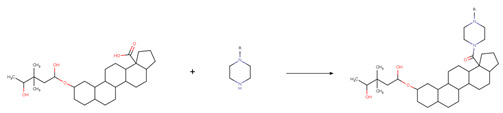
SMIRKS 2.3	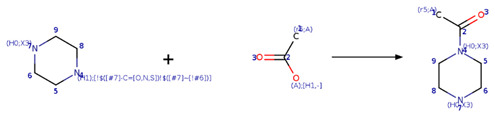 [#6:9]-1-[#6:8]-[#7H1;!$([#7]-C=[O,N,S])!$([#7]~[!#6]):4]-[#6:5]-[#6:6]-[#7;H0X3:7]-1.[#6;A;r5:1][#6:2]([#8;A;H1,-])=[O:3]>>[#6;A;r5:1][#6:2](=[O:3])-[#7;H0X3:4]-1-[#6:5]-[#6:6]-[#7;H0X3:7]-[#6:8]-[#6:9]-1

**Table 3:** Functional groups using SMARTS notation to filter fragments from natural products.

Functional Groups	SMARTS
Aliphatic alcohol (cyclohexanol)	[#8;H1]-[#6]-1-[#6]-[#6]-[#6]-2-[#6](-[#6]-[#6]-[#6]-3-[#6]-4-[#6]-[#6]C5([#6]-[#6]-[#6]-[#6]5-[#6]-4-[#6]-[#6]-[#6]-2-3)[#6]([#8;H1])=O)-[#6]-1
2,2-dimethyl succinic acid	[#6]C([#6])([#6]-[#6](-[#8])=O)[#6](-[#8])=O
piperazine	[#6;H2;X4]1-[#6;H2;X4][#7;X3;!H1][#6;H2;X4]-[#6;H2;X4][#7;H1;X3]1
1,2-diaminoethane	[#7;H1;X3][#6;H2;X4][#6;H2;X4][#7;H2;X3]
1,3-diaminopropane	[#7;H1;X3][#6;H2;X4][#6;H2;X4][#6;H2;X4][#7;H2;X3]
Cyclic system skeleton derived from betulinic acid	[#6]1-[#6]-[#6]-[#6]2-[#6](-[#6]-1)-[#6]-[#6]-[#6]1-[#6]-2-[#6]-[#6]-[#6]2-[#6]3-[#6]-[#6]-[#6]-[#6]-3-[#6]-[#6]-[#6]-1-2

**Table 4:** Properties of pharmaceutical relevance of FDA-approved HIV-1 protease inhibitors.

Parent Molecule	SlogP	MW	HBD	HBA	TPSA	RB
Amprenavir	2.40	505.22	4	9	131.19	11
Atazanavir	4.21	704.39	5	13	171.22	14
Darunavir	2.38	547.24	4	10	140.42	11
Fosamprenavir ^a^	2.69	585.19	4	12	174.56	13
Indinavir	2.87	613.36	4	9	118.03	11
Lopinavir	4.33	628.36	4	9	120.00	15
Nelfinavir	4.75	567.31	4	7	101.90	9
Ritonavir	5.91	720.31	4	11	145.78	17
Saquinavir	3.09	670.38	6	11	166.75	12
Tipranavir	6.70	602.21	1	7	102.43	11
Minimum ^a^	2.40	505.20	1	7	101.90	9
Maximum ^a^	6.70	720.30	6	13	174.60	17

^a^ Maximum and minimum values for each property.

## Data Availability

All datasets used in this study are available on https://figshare.com/s/ceb58d58e8f5585ce67e (accessed on 5 November 2021). TMAP_chemical_space_visualization.html; All_fragments_COCONUT_V4_184769.csv; HIV-protease_inhibitors_from_ChemDiv.csv; HIV_protease_inhibitors_from_COCONUT.csv; HIV_protease_inhibitors_from_Enamine.csv; FDA_APPROVED_DRUGS.csv; HIV_PROTEASE_INHIBITORS.csv; ADMETOX profiling_pkCSM.csv; ADMETOX profiling_swissadme.csv. Code used for generated new chemical compounds from chemical fragments are available on https://github.com/DIFACQUIM/De-novo-desing-of-HIV-1-inhibitors (accessed on 5 November 2021).

## Supplementary Materials

The following are available online at https://www.mdpi.com/article/10.3390/biom11121805/s1, Figure S1: Convex hull area from PCA based on physicochemical properties of new chemical compounds generated and two compound reference libraries. Table S1: Summary of fingerprint-based structural diversity of new chemical compounds generated from COCONUT, ChemDiv, and Enamine fragments, and two compound reference libraries. Figure S2: Box-whisker plots of physicochemical properties of FDA-approved drugs (blue), FDA-approved HIV-1 protease inhibitors (purple), and new chemical compounds generated from COCONUT (orange), ChemDiv (red), and Enamine (green) fragment libraries, before applying physicochemical properties filtering. Table S2: Summary of the descriptive statistics of SlogP. Table S3: Summary of the descriptive statistics of MW. Table S4: Summary of the descriptive statistics of RB. Table S5: Summary of the descriptive statistics of TPSA. Table S6: Summary of the descriptive statistics of HBA. Table S7: Summary of the descriptive statistics of HBD. Table S8: Summary of the descriptive statistics of SAscore. Table S9: Summary of the descriptive statistics of solubility (Silicos-IT LowSw). Table S10: Summary of the descriptive statistics of lipophilicity (Consensus Log P). Table S11: Summary of the descriptive statistics of HIA. Table S12: Summary of the descriptive statistics of BBB permeability. Table S13: Percentage of compounds that are P-glycoprotein substrate, P-glycoprotein I inhibitor, and P-glycoprotein II inhibitor. Table S14: Percentage of compounds that inhibit the main cytochromes, CYP1A2, CYP2C19, CYP2C9, CYP2D6, CYP3A4. Table S15: Summary of the descriptive statistics of total clearance. Table S16: Summary of the descriptive statistics of toxicity descriptors.
