# Supplementary material for: Towards the De Novo Design of HIV-1 Protease Inhibitors Based on Natural Products
Source: Biomolecules. 2021 Dec 1;11(12):1805. doi: 10.3390/biom11121805 (PMC8698858; doi:10.3390/biom11121805)
Supplement: Supplementary file 1 [file biomolecules-11-01805-s001.zip › biomolecules-1473232-supplementary.pdf]

Supplementary material

Towards the de novo Design of HIV-1 Protease Inhibitors based on Natural Products

Ana L. Chávez-Hernández, K. Eurídice Juárez-Mercado, Fernanda I. Saldívar-González, and José L. Medina-Franco

Contents

		Page
**Figure S1**	Convex hull area from PCA based on physicochemical properties of new chemical compounds generated and two compound reference libraries.	S2
**Table S1**	Summary of fingerprint-based structural diversity of new chemical compounds generated from COCONUT, ChemDiv, and Enamine fragments, and two compound reference libraries.	S3
**Figure S2**	Box-whisker plots of physicochemical properties of FDA-approved drugs (blue), FDA-Approved HIV-1 protease inhibitors (purple), and new chemical compounds generated from COCONUT (orange), ChemDiv (red), and Enamine (green) fragment libraries, before applying physicochemical properties filtering.	S4
**Table S2**	Summary of the descriptive statistics of SlogP.	S5
**Table S3**	Summary of the descriptive statistics of MW.	S5
**Table S4**	Summary of the descriptive statistics of RB.	S5
**Table S5**	Summary of the descriptive statistics of TPSA.	S6
**Table S6**	Summary of the descriptive statistics of HBA.	S6
**Table S7**	Summary of the descriptive statistics of HBD.	S6
**Table S8**	Summary of the descriptive statistics of SAscore.	S7
**Table S9**	Summary of the descriptive statistics of solubility (Silicos-IT LowSw).	S7
**Table S10**	Summary of the descriptive statistics of lipophilicity (Consensus Log P).	S7
**Table S11**	Summary of the descriptive statistics of HIA.	S8
**Table S12**	Summary of the descriptive statistics of BBB permeability.	S8
**Table S13**	Percentage of compounds that are P-glycoprotein substrate, P-glycoprotein I inhibitor, and P-glycoprotein II inhibitor.	S8
**Table S14**	Percentage of compounds that inhibit the main cytochromes, CYP1A2, CYP2C19, CYP2C9, CYP2D6, CYP3A4.	S9
**Table S15**	Summary of the descriptive statistics of total clearance.	S9
**Table S16**	Summary of the descriptive statistics of toxicity descriptors.	S9

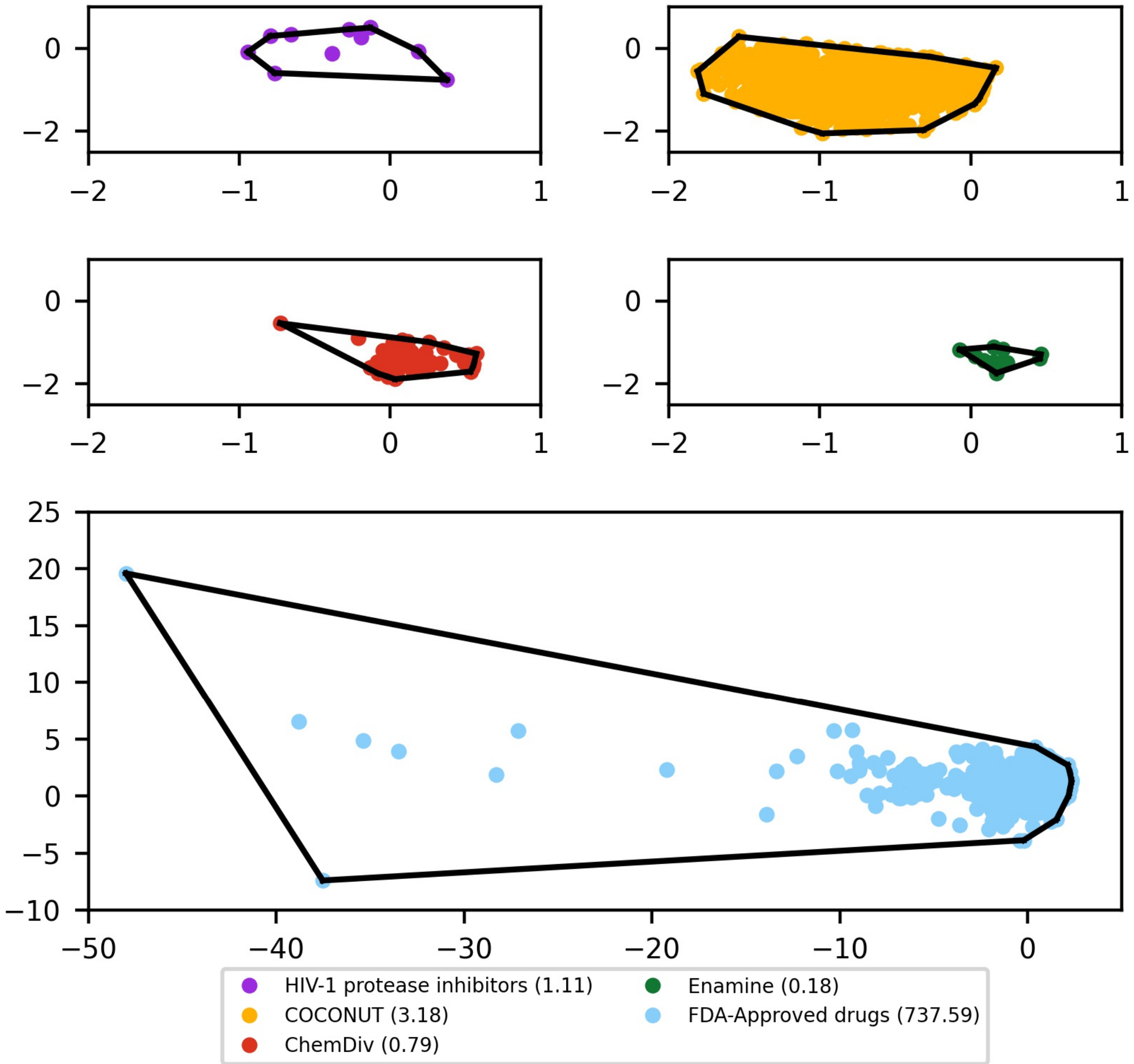

Figure S1. Convex hull area from PCA based on physicochemical properties of new chemical compounds generated and two compound reference libraries. Compound reference libraries represented in colors: FDA-approved drugs (blue), and FDA-approved HIV-1 protease inhibitors (purple). Likewise for new chemical compounds generated from COCONUT (orange), ChemDiv (red), and Enamine (green) fragment libraries. Convex Hull area represented in parenthesis.

Table S1. Summary of fingerprint-based structural diversity of new chemical compounds generated from COCONUT, ChemDiv and Enamine fragments, and two compound reference libraries, FDA-approved drugs, and FDA-approved HIV-1 protease inhibitors. [a] Median similarity.

Data set	Morgan2 [a] (1024-bits)	MACCS Keys[a] (166-bits)
COCONUT	0.605	0.817
Enamine	0.682	0.823
ChemDiv	0.676	0.821
FDA-Approved drugs	0.096	0.293
FDA-Approved HIV-1 protease inhibitors	0.253	0.558

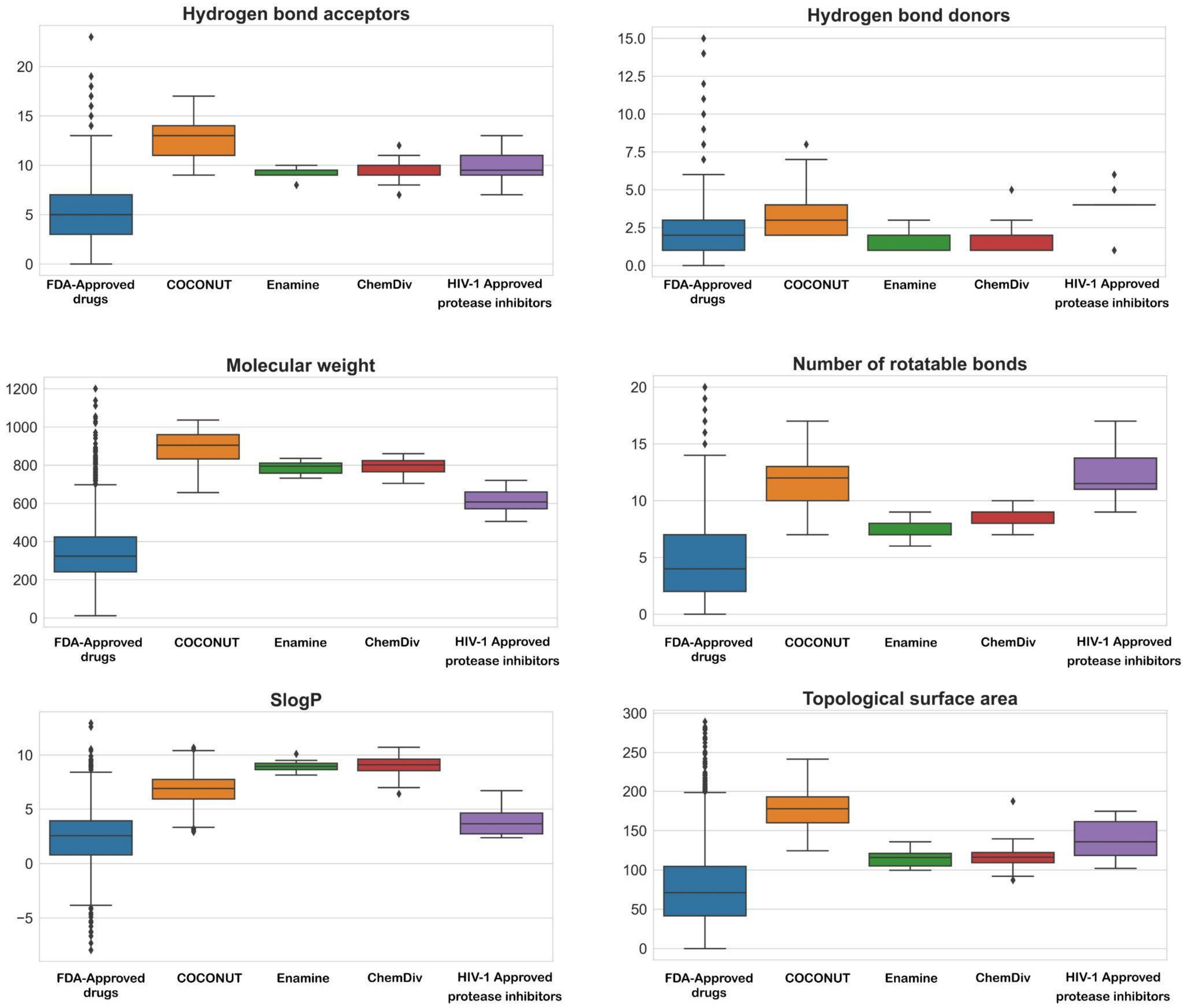

Figure S2: Box-whisker plots of physicochemical properties of FDA-approved drugs (blue), FDA-approved HIV-1 protease inhibitors (purple), and new chemical compounds generated from COCONUT

(orange), ChemDiv (red), and Enamine (green) fragment libraries, before applying physicochemical properties filtering.

Table S2: Summary of the descriptive statistics of SlogP.

Data Set	Count	Mean	Std	Min	25%	50%	75%	Max
COCONUT	352.0	5.87	0.64	3.77	5.50	6.00	6.38	6.69
ChemDiv	1.0	6.40	0.00	6.40	6.40	6.40	6.40	6.40
FDA-Approved drugs	2133.0	2.34	2.53	-8.81	0.88	2.56	3.94	12.94
HIV-1 protease inhibitors	10.0	3.93	1.51	2.38	2.73	3.65	4.64	6.70

Table S3. Summary of the descriptive statistics of MW.

Data set	Count	Mean	Std	Min	25%	50%	75%	Max
COCONUT	352.0	810.46	69.30	656.44	753.44	810.97	864.52	998.63
ChemDiv	1.0	737.47	0.00	737.47	737.47	737.47	737.47	737.47
FDA-Approved drugs	2133.0	349.22	159.99	12.00	245.15	327.22	428.16	1201.84
HIV-1 protease inhibitors	10.0	614.40	68.55	505.22	571.78	607.78	659.88	720.31

Table S4: Summary of the descriptive statistics of RB.

Data set	Count	Mean	Std	Min	25%	50%	75%	Max
COCONUT	352.0	10.43	1.83	7.0	9.0	10.0	12.00	15.0
ChemDiv	1.0	10.00	0.00	10.0	10.0	10.0	10.00	10.0
FDA-Approved drugs	2133.0	4.88	3.68	0.0	2.0	4.0	7.00	20.0
HIV-1 protease inhibitors	10.0	12.40	2.37	9.0	11.0	11.5	13.75	17.0

Table S5: Summary of the descriptive statistics of TPSA.

Data set	Count	Mean	Std	Min	25%	50%	75%	Max
COCONUT	352.0	164.99	16.24	124.45	153.55	167.68	177.56	198.54
ChemDiv	1.0	187.47	0.00	187.47	187.47	187.47	187.47	187.47
FDA-Approved drugs	2133.0	79.43	51.83	0.00	41.57	71.43	104.70	286.50
HIV-1 protease inhibitors	10.0	137.23	27.21	101.90	118.52	135.80	161.51	174.56

Table S6: Summary of the descriptive statistics of HBA.

Data set	Count	Mean	Std	Min	25%	50%	75%	Max
COCONUT	352.0	11.64	1.19	9.0	11.0	12.0	13.0	13.0
ChemDiv	1.0	12.00	0.00	12.0	12.0	12.0	12.0	12.0
FDA-Approved drugs	2133.0	5.57	3.43	0.0	3.0	5.0	7.0	23.0
HIV-1 protease inhibitors	10.0	9.80	1.99	7.0	9.0	9.5	11.0	13.0

Table S7: Summary of the descriptive statistics of HBD.

Data set	Count	Mean	Std	Min	25%	50%	75%	Max
COCONUT	352.0	3.17	0.99	2.0	2.0	3.0	4.0	7.0
ChemDiv	1.0	5.00	0.00	5.0	5.0	5.0	5.0	5.0
FDA-Approved drugs	2133.0	2.08	1.97	0.0	1.0	2.0	3.0	15.0
HIV-1 protease inhibitors	10.0	4.00	1.25	1.0	4.0	4.0	4.0	6.0

Table S8: Summary of the descriptive statistics of SAscore.

Data set	Count	Mean	Std	Min	25%	50%	75%	Max
COCONUT	352.0	5.85	0.39	5.40	5.56	5.72	6.03	7.33
ChemDiv	1.0	5.54	0.00	5.54	5.54	5.54	5.54	5.54
FDA-Approved drugs	2133.0	3.28	1.17	1.06	2.43	3.01	3.93	8.49
HIV-1 protease inhibitors	10.0	4.03	0.22	3.48	3.98	4.06	4.17	4.24

Table S9: Summary of the descriptive statistics of solubility (Silicos-IT LowSw).

Data set	Count	Mean	Std	Min	25%	50%	75%	Max
COCONUT_ChemDiv	252.0	-6.48	1.16	-9.49	-7.19	-6.45	-5.60	-4.07
FDA-Approved drugs	2243.0	-4.38	3.14	-19.66	-6.20	-4.34	-2.44	12.92
HIV-1 protease inhibitors	10.0	-8.09	2.18	-11.13	-9.88	-8.49	-5.98	-5.12

Table S10: Summary of the descriptive statistics of lipophilicity (Consensus Log P).

Data set	Count	Mean	Std	Min	25%	50%	75%	Max
COCONUT_ChemDiv	252.0	4.67	0.61	3.00	4.28	4.70	5.10	5.92
FDA-Approved drugs	2243.0	2.14	2.31	-11.57	0.82	2.36	3.58	15.93
HIV-1 protease inhibitors	10.0	3.58	1.36	1.45	2.56	3.50	4.38	5.90

Table S11: Summary of the descriptive statistics of HIA.

Data set	Count	Mean	Std	Min	25%	50%	75%	Max
COCONUT_ ChemDiv	252.0	66.77	8.99	27.33	63.11	67.90	72.68	82.37
FDA-Approved drugs	2208.0	77.33	26.94	0.00	69.74	90.60	94.54	100.00
HIV-1 protease inhibitors	9.0	66.27	9.76	53.06	62.64	64.42	65.52	88.32

Table S12: Summary of the descriptive statistics of BBB permeability.

Data set	Count	Mean	Std	Min	25%	50%	75%	Max
COCONUT_ ChemDiv	251.0	-1.21	0.20	-1.96	-1.34	-1.21	-1.06	-0.81
FDA-Approved drugs	2208.0	-0.60	1.34	-33.79	-1.12	-0.38	0.10	1.63
HIV-1 protease inhibitors	9.0	-1.12	0.39	-1.72	-1.36	-1.25	-0.75	-0.62

Table S13: Percentage of compounds that are P-glycoprotein substrate, P-glycoprotein I inhibitor, and P-glycoprotein II inhibitor.

Data Set	P-Glycoprotein Substrate	P-Glycoprotein I Inhibitor	P-Glycoprotein II Inhibitor
COCONUT_ChemDiv	96.03%	0.00%	82.94%
FDA-Approved drugs	62.95%	29.85%	23.73%
HIV-1 protease inhibitors	100.00%	100.00%	66.67%

Table S14: Percentage of compounds that inhibit the main cytochromes, CYP1A2, CYP2C19, CYP2C9, CYP2D6, and CYP3A4.

Data Set	CYP1A2 Inhibitor	CYP2C16 Inhibitor	CYP2C9 Inhibitor	CYP2D6 Inhibitor	CYP3A4 Inhibitor
COCONUT_ChemDiv	0.00%	0.00%	0.00%	0.00%	0.00%
FDA-Approved drugs	26.77%	16.58%	11.96%	16.62%	18.75%
HIV-1 protease inhibitors	0.00%	33.33%	33.33%	0.00%	88.89%

Table S15: Summary of the descriptive statistics of total clearance.

Data Set	Count	Mean	Std	Min	25%	50%	75%	Max
COCONUT_ChemDiv	252.0	-0.57	0.28	-1.34	-0.77	-0.62	-0.34	0.13
FDA-Approved drugs	2208.0	0.53	0.67	-13.94	0.27	0.59	0.85	3.44
HIV-1 protease inhibitors	9.0	0.50	0.22	0.20	0.38	0.49	0.56	0.94

Table S16: Summary of the descriptive statistics of toxicity descriptors.

Data Set	hERG I Inhibitor	hERG II Inhibitor	Hepatotoxicity	AMES Toxicity
COCONUT_ChemDiv	0.00%	1.19%	72.22%	0.00%
FDA-Approved drugs	2.31%	33.65%	47.42%	16.76%
HIV-1 protease inhibitors	0.00%	77.78%	100.00%	0.00%
